# Roadmap to Local Tumour Growth: Insights from Cervical Cancer

**DOI:** 10.1038/s41598-019-49182-1

**Published:** 2019-09-04

**Authors:** Hans Kubitschke, Benjamin Wolf, Erik Morawetz, Lars-Christian Horn, Bahriye Aktas, Ulrich Behn, Michael Höckel, Josef Käs

**Affiliations:** 1 Peter Debye Institute for Soft Matter Physics, Leipzig University, Leipzig, Germany; 2 Department of Gynecology, Women’s and Children’s Centre, University Hospital Leipzig, Leipzig, Germany; 3 Leipzig School of Radical Pelvic Surgery, Leipzig University, Leipzig, Germany; 4 Division of Gynecologic, Breast and Perinatal Pathology, University Hospital Leipzig, Leipzig, Germany; 5 Institute of Theoretical Physics, Leipzig University, Leipzig, Germany

**Keywords:** Cancer models, Cervical cancer, Surgical oncology, Urological cancer

## Abstract

Wide tumour excision is currently the standard approach to surgical treatment of solid cancers including carcinomas of the lower genital tract. This strategy is based on the premise that tumours exhibit isotropic growth potential. We reviewed and analysed local tumour spreading patterns in 518 patients with cancer of the uterine cervix who underwent surgical tumour resection. Based on data obtained from pathological examination of the surgical specimen, we applied computational modelling techniques to simulate local tumour spread in order to identify parameters influencing preferred infiltration patterns and used area-proportional Euler diagrams to detect and confirm ordered patterns of tumour spread. Some anatomical structures, e.g. tissues of the urinary bladder, were significantly more likely to be infiltrated than other structures, e.g. the ureter and the rectum. Computational models assuming isotropic growth could not explain these infiltration patterns. Introducing ontogenetic distance of a tissue relative to the uterine cervix as a parameter led to accurate predictions of the clinically observed infiltration likelihoods. The clinical data indicates that successive infiltration likelihoods of ontogenetically distant tissues are nearly perfect subsets of ontogenetically closer tissues. The prevailing assumption of isotropic tumour extension has significant shortcomings in the case of cervical cancer. Rather, cervical cancer spread seems to follow ontogenetically defined trajectories.

## Introduction

Cancer of the breast, prostate, colorectum, lung, and cervix uteri are among the most commonly diagnosed malignant tumours worldwide^1^. All of these cancers are solid tumours and surgery or (chemo-) radiotherapy are currently the only available options for curative loco-regional treatment^2–5^. Generally, the goal of surgery should be to minimize loco-regional recurrence rates without increasing operative morbidity unnecessarily. To accomplish these objectives, the surgeon is faced with the following central questions: (I) Which tissues are at risk for both visible or occult tumour infiltration and need to be removed? (II) Which tissues can be safely spared, thus minimizing treatment-related morbidity? (III) Which locally advanced tumours can still be submitted to surgical treatment? Conventionally, the answer to these questions has been based on two assumptions: First, that local tumour growth is unpredictable and therefore can occur in any direction, and second, that a microscopically invisible tumour front precedes the identifiable tumour margin. These dogmas of local tumour growth are reflected in the surgical treatment strategy of wide excision. Hereby, a metrically defined circumferential safety margin of healthy tissue is excised around the tumour with the goal of removing all (occult) tumour cells that are thought to permeate this region. Even though a single malignant neoplasm usually does not display isotropic growth, it is thought to have isotropic growth *potential*. As the actual direction of local cancer growth in a given case is assumed to be stochastic, the average growth pattern of multiple tumours is expected to be isotropic. The tissue at risk for infiltration of occult tumour cells is therefore currently defined as an isotropic tissue rim which is removed by wide excision.

However, increasing evidence points to fundamental flaws in the wide excision strategy. Even when adequate wide excision margins are obtained surgically, local relapse rates remain high^6,7^. Furthermore, the width of resection margins would be expected to be one of the most important determinants of local tumour control. However, clinical data does not support this concept as has been demonstrated for several solid tumours, and reports from multiple investigations on resection margins provide conflicting results with some studies indicating that the width of resection margins is an important prognostic indicator while others fail to show an effect on local tumour recurrence and survival^8–11^. Imaging modalities are currently not capable of identifying single occult tumour cells that might precede the cancer invasion front. What surgeons need to know, therefore, is which tissues are at risk for *potential* infiltration of occult tumour cells and need to be removed to ensure oncological safety. Likewise, tissues that have a low probability of tumour infiltration should be spared with the goal of decreasing treatment-related morbidity. As a proxy for other solid tumours, we analysed data of cancer of the uterine cervix to demonstrate that local tumour spread is not isotropic but follows predictable growth patterns.

Based on clinical experience during the last two decades^11–14^, we hypothesized that these anisotropic growth patterns would fit to an ontogenetic distance map, with tissues exhibiting a lesser ontogenetic distance to the uterine cervix having a higher probability of being infiltrated by the tumour than those of greater ontogenetic distance. The corresponding null-hypothesis would predict that the observed tumour infiltration patterns could be explained by physical distance alone, which is a function of metric distance and physical tissue type (i.e. adipose tissue or muscle). In this investigation, we use data derived from detailed pathology reports of patients who underwent surgical treatment for cervical cancer at our institution. Based on the results from pathological examination of all tissues removed during surgery, we determine in each case which pelvic structures are infiltrated by cancer. We then use area-proportional Euler diagrams and computational tumour growth simulations to determine whether ontogenetic tissue distance (relative to the uterine cervix) is a better parameter for the prediction of cancer infiltration than microenvironmental (physical) distance. The assumption of predictable and ordered rather than stochastic tumour growth patterns could form the basis of a roadmap for local tumour growth which might help surgeons or radiotherapists identify tissues at high risk for tumour infiltration.

## Results

### Pathological and ontogenetic characterization

To determine the clinical relevance of our findings and to facilitate their clinical application, we characterized our patient sample according to standard criteria; general patient- and tumour characteristics are compiled in Table 1. Our cohort contained a large number of locally advanced cases represented by pathological tumour stages 1b2 and higher in 50.2% of cases. In addition, 33.2% of all patients exhibited regional lymph node metastasis indicating advanced disease. The majority (74.9%) of all cases were squamous cell carcinomas.

**Table 1:** Patient and tumour data:

Parameter	n	%
Total n = 518 Age (median, IQR)	45 (37–54)	
**Histology**		
Squamous cell carcinoma	388	74.9
Adenocarcinoma	103	19.9
Adenosquamous carcinoma	23	4.4
Small cell carcinoma	4	0.8
**Type of surgery** ^**a**^		
TMMR	437	84.3
EMMR	49	9.5
LEER	32	6.2
**Lymphovascular space invasion**		
Yes	360	69.5
No	152	29.3
Unknown^b^	6	1.2
**Blood vessel invasion**		
Yes	66	12.7
No	446	86.1
Unknown^b^	6	1.2
**Grading**		
G1	75	14.5
G2	253	48.8
G3	185	35.7
Unknown^b^	5	1.0
**FIGO Stage**		
IA2 – IB1	267	51.5
IB2 – IIB	219	42.3
>IIB	32	6.2
**Pathological tumour stage**		
1a2 – b1	258	49.8
1b2 – 2b	249	48.1
>2b	11	2.1
**Pelvic lymph node metastasis present**		
Yes	172	33.2
No	346	66.8
**Paraaortic lymph node metastasis present** ^**c**^		
Yes	43	8.3
No	475	91.7

^a^Total mesometrial resection (TMMR) is a surgical resective procedure by which the adult derivatives of the embryonal Müllerian Anlage (uterus [cervix and corpus], the Fallopian tubes, the proximal vagina, and the vascular and ligamentous mesometrium) are removed. In extended mesometrial resection (EMMR), tissues in addition to those removed by TMMR are resected. Depending on the tumour extension and the surgical situation, this may include the distal ureter and its supporting structure (the mesureter), parts of the urinary bladder wall and its adventitia, the distal vagina, or parts of the peritoneum. In addition to the aforementioned tissues, laterally extended endopelvic resection (LEER) includes removal of pelvic wall structures such as parts of the obturator muscle, the complete bladder with its adventitia and supporting structures, the rectum with its supporting structures, or both.

^b^In six cases the entire tumour had been removed by local excision (conisation) for a suspected cervical intraepithelial neoplasia prior to the operation and external pathology reports did not include information on lympho-vascular space invasion. In four of these cases, information on tumour grade was also not available.

^c^Periaaortic lymph node status was assessed histopathologically in 100 patients (19.3% of all patients). In the remaining cases paraaortic lymph node status was assumed negative because of histopathologically proven tumour free pelvic nodes and the absence of tumour infiltration of the uterine corpus. In this situation, metastasis in the periaortic region is unlikely.

**Figure 1:**
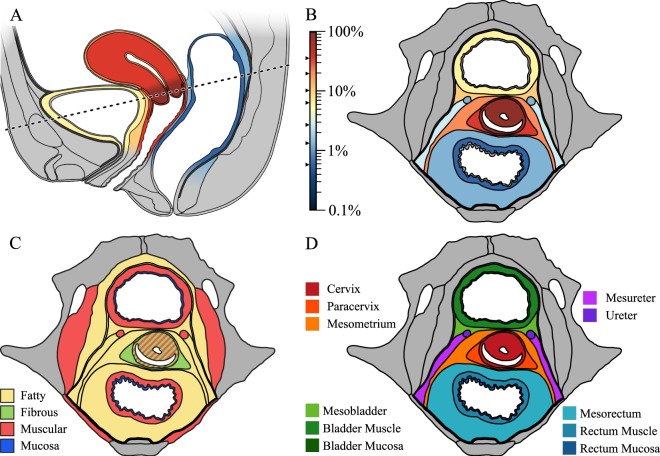
Anatomical maps of the female pelvis at the level of the uterine cervix. Tumour infiltration probability of 518 cervical cancer patients is depicted in the sagittal plane, (**A**) and the transversal plane, (**B**) respectively as heat maps. Despite the circumstance that bladder, rectum, and ureter are in similar metric proximity to the uterine cervix, the infiltration probabilities for each tissue at the time of surgical treatment is different. (**C**) Depicts the color-coded tissue types. Notably, the uterine cervix is surrounded by fibrous-fatty connective tissue in all directions. (**D**) Displays tissue compartments surrounding the uterine cervix classified according to their ontogenetic distance relative to the cervix. These compartments are separated by fine collagen lamellae which can be surgically dissected.

Detailed two-dimensional anatomical maps in the sagittal and transversal plane of the pelvis were drawn and tumour infiltration probabilities for different tissues and tissue compartments adjacent to the uterine cervix were mapped to these drawings (Fig. 1A,B) based on pathological-identified infiltrated structures. In addition, different tissue types (e.g. fatty and muscular tissue) were identified and charted in the anatomical maps (Fig. 1C). Furthermore, anatomical tissues were classified and mapped according to their ontogenetic origin (Fig. 1D, described below). Tissues which are ontogenetically close to each other and constitute a functional unit were labelled with similar colours (reddish, greenish, purplish and blueish; Fig. 1D**)**. A detailed description of the ontogenetic anatomy that exhibits some differences to conventional anatomy is available in the methods section.

### Tumour size and shape characteristics

**Figure 2:**
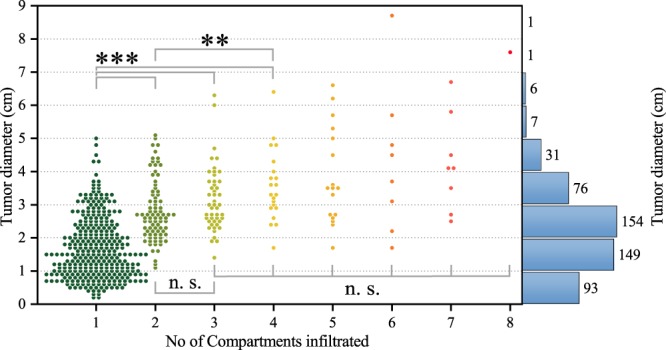
Scatter plot showing the number of infiltrated anatomical compartments with corresponding tumour diameter. An overall tumour diameter histogram is drawn on the right side. While the tumour size correlates with the number of infiltrated compartments for small sizes, especially when three compartments or less are infiltrated (Spearman’s rank order correlation of *ρ* = 0.599), the data set of tumour diameters infiltrating more than two structures is only weakly correlated (Spearman’s rank order correlation of *ρ* = 0.340). Asterisks indicate the level of significance of the two-sided Kolmogorov–Smirnov test with *p* < 0.05, *p* < 0.01, *p* < 0.001 for one, two and three asterisks, respectively. Branched indicators for significance denote significance between all pairs of included measurements. Importantly, however, there is a wide distribution of tumour sizes for each compartment infiltrated.

We compared the gross tumour diameter with the number of tumour-infiltrated tissue compartments categorised in Fig. 1D. We found a reasonable correlation for small tumours only where three or less tissue compartments were infiltrated (Fig. 2, Spearman’s rank order correlation of *ρ* = 0.599). In contrast, we observed only a weak correlation between the two parameters when a higher number, *n* ≥ 3, of structures were infiltrated (*ρ* = 0.340). A very similar picture was seen when grouping the tumour data in two sets, one set with tumour diameter ≤4 cm in greatest dimension (FIGO staging IB1/IIA1) and one with >4 cm (FIGO staging IB2/IIA2). The correlation between infiltrated structures and tumour size for small tumours below 4 cm was again quite reasonable (Spearman’s rank order correlation of *ρ* = 0.584), whereas the same correlation was weaker for larger tumours (*ρ* = 0.389).

**Figure 3:**
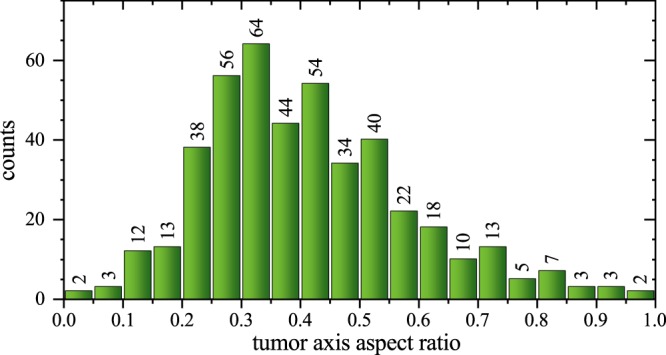
Histogram depicting the distribution of the aspect ratios of our cervical cancer cohort (*n* = 518). A large fraction of tumours has an aspect ratio profoundly deviating from 1 illustrating that the tumours are not of spherical growth.

In Fig. 3 the tumour shape aspect ratio of short to long axis, i.e. the Diameter (Short axis)/Diameter (long axis), is shown which was derived from the metric 3-dimensional measures of the tumours. An aspect ratio of 1 would represent a spherical and 0 a planar (oblate) or needle-shaped (prolate) tumour. We found an average aspect ratio of approx. 0.4.

### Descriptive modelling of tumour infiltration

**Figure 4:**
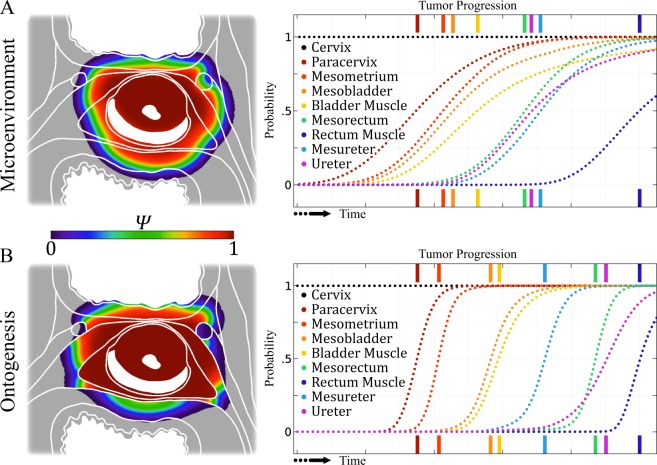
Illustration of the infiltration probability of tissue compartments over time. The model is based on a two-dimensional Fisher-Kolmogorov equation (*n* = 1, *D* = 1.7 m^2^/*s*, *r*_muscle_ = 0.0047 · 1/day, *r*_fatty_ = 0.019 · 1/day, *r*_fibrous_ = 0.014 · 1/day). The left figures display the color-coded cancer cell probability density *Ψ*. Histopathological data of tumour infiltration displays distinct separation of infiltration probability of cervix and bladder associated compartments (reddish and yellowish lines) and rectum and ureter associated compartments (greenish and blueish lines). In the microenvironmental model, (**A**) which only considers tissue-type dependent varying diffusion and tumour cell proliferation rates, the invasion probability of spatially close compartments increases with tumour progression almost concurrently. A clear separation of tumour stages is hampered because the invasion probability curves are significantly overlapping. The microenvironmental model does not provide adequate predictions for compartment infiltration. In the ontogenetic model, (**B**) tissue compartment boundaries were modelled as additional resistive passive barriers which limit cancer cell migration, as described in the methods section. Invasion of spatially close compartments happens stepwise, as seen in clinical data. The stepwise order and invasion likelihood of infested compartments can only be correctly represented when considering ontogenetic segregation of tissue compartments.

We ran two different *in silico* simulations, each incorporating one of the two models. Results from the tumour growth simulations are shown in Fig. 4. We found that clinical data of tumour infiltration displays a distinct separation of the infiltration probabilities of cervix and bladder associated tissue compartments, and furthermore, of rectum and ureter associated compartments. In the physical microenvironmental model which only considers tissue-type dependent diffusion variation and tumour cell proliferation rates, the invasion probability of spatially close compartments increases with tumour progression almost concurrently. This is displayed by the broad overlapping tumour infiltration probability evolution in Fig. 4A around the inflection points.

In the ontogenetic model (Fig. 4B), tissue compartment boundaries were modelled as additional resistive barriers which limit cancer cell migration. Tissues that are ontogenetically close to the cervix are always invaded before an ontogenetically more distant compartment is infiltrated, e.g. the bladder compartment, the ureter, or the rectum compartment. There is a distinct separation of the infiltration probabilities around the inflection point.

### Stepwise tumour infiltration

**Figure 5:**
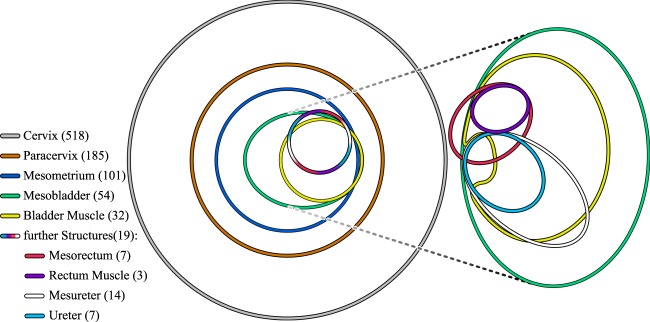
Area-proportional Euler diagram displaying infiltration of endopelvic (sub-) compartments of 518 pooled cervical cancer cases. The patients underwent either Total Mesometrial Resection (TMMR), Extended Mesometrial Resection (EMMR) or Laterally Extended Endopelvic Resection (LEER) as local excisional procedures. The areas of the ellipses are proportional to the total number of reported cases of the corresponding infiltrated compartments and sub-compartments. Overlying areas of two or more ellipses represent cases where several compartments are infiltrated. The intersectional area is also proportional to the number of cases in which multiple compartments are infiltrated. A smaller ellipse that is completely located inside a larger one is therefore a true subset of the larger ellipse. For example, mesometrial infiltration incidents (blue) only occur if the paracervix (orange) has been previously infiltrated. Notably, up to the infiltration of the bladder compartment, the tissue involvement is practically following a stepwise progression which is reflected in ellipses being true subsets of enclosing larger ellipses. Exact definitions of the anatomical structures described here can be found in the methods section.

Tumour infiltration data was gathered from the pathology reports containing the findings of the examination of the surgically resected tissues. Area-proportional Euler diagrams^15–17^ of the histopathological data of tumour infiltration were drawn in Fig. 5. The Euler diagram illustrates that up to the ellipse which displays bladder muscle infiltration (yellow), the numbers of patients with tumour infiltration of tissue compartments with successively increasing ontogenetic distance from the uterine cervix are nearly perfect subsets of the numbers of patients with infiltration of ontogenetically closer compartments. For example, infiltration of the paracervix and the mesometrium was present in 35.7% and 19.4% of cases. The bladder mesentery and the bladder itself were involved by the tumour in 10.4% and 6.2% of cases, the mesureter and ureter in 2.7% and 1.4%, respectively. The rectum, 0.6%, and the mesorectum, 1.4%, showed the lowest likelihood of tumour infiltration. There was only one out of 54 cases of bladder mesentery and bladder muscle infiltration without mesometrial involvement (1.9%, green, yellow and blue). Furthermore, we found only one case where the mesorectum was infiltrated without concurrent mesobladder involvement (green and amaranth). Further information about the anatomical structures described here can be found in the methods section and separate diagrams for squamous cell and non-squamous cell carcinomas are included in the Supplement S3.

## Discussion

In this work, we demonstrate that local tumour growth potential of cervical cancer is not isotropic and that the infiltration probability differs greatly among different anatomical structures and compartments in close proximity to the uterine cervix.

As a first step to investigate whether tumour growth displays isotropic patterns, we compared the gross tumour diameter with the number of tumour-infiltrated tissue compartments (Fig. 1D). If all surrounding compartments displayed an equal infiltration likelihood, tumour size would be expected to correlate clearly with the number of infiltrated compartments. However, we found a reasonable correlation for small tumours (less or equal to three compartments infiltrated) but only a weak correlation for larger tumours (infiltration of more than three compartments). Notably, while tumours with diameter of 1.5 cm and lower are practically bound to the uterine cervical compartment, larger tumours do not necessarily invade more compartments. Taken together, in contrast to what our null-hypothesis would have predicted, we found that tumours of one size (diameter) can infiltrate a varying number of compartments indicating that local tumour growth does not occur in a purely isotropic manner.

Consequentially, we analysed whether the average tumour shape aspect ratio in our cohort was close to spherical as would have been predicted by our null-hypothesis assuming similar infiltration likelihoods of all surrounding tissues. Contrarily to an idealised, isotropically growing tumour – which is assumed in a wide excision strategy and would have an aspect ratio of very close to 1 – we found that the majority of aspect ratios significantly diverge from 1 with an average aspect ratio of approx. 0.4. While the localisation and shape of the initially formed tumour may influence the shape of the growing tumour, the surgically resected and pathologically analysed tumours presented in Figs 2 and 3 are orders of magnitudes larger in volume. Thus, under the premise of diffusive undirected tumour growth, the shape and aspect ratio of the tumour should exhibit a trend towards a more spherically shaped tumour, which is not observed in the data set. The strong deviation of the found aspect ratio from 1 indicates that cervical cancer tumour growth is not isotropic on average on large scales compared to other tumour entities such as glioblastomas and breast cancers, which grow in a more uniform microenvironment and display a profoundly more spherical aspect ratio close to 0.7^18–20^.

To further investigate whether ontogenetic distance is a better parameter than physical (i.e. microenvironmental) distance to predict local tumour growth patterns, we ran *in silico* simulations for a physical microenvironmental and an ontogenetic model. The microenvironmental model is agnostic to ontogenetic compartment boundaries. The infiltration probabilities of different compartments are vastly overlapping, thus the expected number of cases with, for instance, mesorectum infiltration without mesobladder infiltration should be far beyond a singular case as observed in our cohort. Therefore, the microenvironmental model does not adequately reflect tumour infiltration patterns from a probabilistic point of view and physical distance is not a useful parameter in predicting local tumour growth. In the ontogenetic model, however, invasion of spatially close compartments happens stepwise, which is in accordance with the histopathological data. In detail, the tumour infiltration probabilities of tissues with growing ontogenetic distance from the uterine cervix increase suddenly with distinct onsets, in contrast to the overlapping infiltration probabilities of the microenvironmental model. Our tumour growth modelling data shows that introducing ontogenetic distance of tissues relative to the uterine cervix leads to a significant improvement in predicting the clinically observed tumour growth patterns as compared to models assuming isotropic tumour growth in the context of microenvironmental factors alone (microenvironmental or physical distance). However, some points in each model deserve further discussion:

First, one should consider how differences in the tumour microenvironment might lead to preferential infiltration of specific abutting tissues. Fatty, muscular and fibrous connective tissues all constitute different microenvironments in which tumour cells might behave differently. For example, fatty tissue has been shown to promote of tumour growth^21,22^ (which would translate into an increased cancer cell proliferation rate *r*_*ij*_). However, tumour cells would be expected to exhibit comparable growth and proliferation behaviour in similar tissue types. The fibro-fatty connective tissues surrounding the uterine cervix ranging to the urinary bladder, the rectum, and the ureter are morphologically very similar and therefore cancer should show similar propensities to extend in either direction rendering tumour growth isotropic on average. Furthermore, the supporting (meso-) structures of bladder, ureter and rectum are all composed of fatty tissue, yet are infiltrated at significantly different rates^13^, see Fig. 1C.

Second, during the past decade, there has been an increasing notion that rigidity of the tumour microenvironment plays an important role in directing tumour growth. Extracellular matrix (ECM) stiffness might in fact promote malignant transformation and direct tumour growth along gradients of ECM rigidity, a process termed durotaxis^23,24^. To date, no measurements of stiffness of the tissues surrounding the uterine cervix have been performed; therefore, we could not include tissue stiffness as a parameter in our model. Nonetheless, it seems unlikely that morphologically similar tissues such as the fatty tissue of the bladder mesentery or the rectal mesentery exhibit significantly different degrees of rigidity or rigidity gradients. In fact, morphologically similar cells share important mechanical and regulatory characteristics and respond and adapt to the rigidity of their surroundings in similar ways^23,25–28^. The impact of the microenvironment is also reflected in the active regulation and fine-tuning of the cell’s cytoskeleton via crosslinker and filament concentrations^29–35^ and therefore the resulting mechanical properties of the cells^36–39^. While it cannot be ruled out that the tissue microenvironment may have an influence of the growth direction and anisotropy, it can be argued that the physical microenvironmental factors cannot be the dominant factor in the process.

Third, it should be assessed whether fascial tissue structures, i.e. dense collagen layers between neighbouring tissues which usually coincide with developmental compartment boundaries, impede tumour growth and thus lead to anisotropic permeation patterns. Recently, using a novel slicing and staining technique, Steinke *et al*. have shown dense, parallel aligned collagen layers between abutting pelvic tissue complexes such as the uterine cervix and the rectum or urinary bladder^40^. These collagen layers may halt locally invading tumour cells. In fact, it has previously been shown that low-to-high concentrated collagen interfaces drastically limit cell migration^25,41–43^. The implementation of collagenous tissue boundaries in our model via uniform, passive resistive boundaries – that is, as a simple mechanical barrier – did not sufficiently lead to the clinically observed growth patterns and shape of cervical cancer. It should be noted, however, that the use of strict inter-compartmental barriers in our model predicts a step-wise tumour progression into neighbouring tissue compartments and thus captures one aspect of the clinically observed tumour permeation pattern. It fails, however, to adequately reflect the anatomical distribution of tumour spread. Nonetheless, dense collagen lamellae remain a crucial physical barrier in (cancer) cell migration. Especially the ontogenetic lineage boundary between the mesometrium and mesorectum, as well as the mesureter, is extraordinary resilient against tumour transmigration, whereas the collagen lamella between the mesometrium and bladder compartment seems to be a minor obstacle; an unexpected observation considering that all collagen lamellae are of comparable thickness and composition and thus should display the same migratory resistance. Given the high spatial density of segregated compartments and therefore proximity of compartment boundaries, the tumour growth is influenced and shaped by these compartment boundaries, described in previous findings^11–14^. However, when interpreting the cancer cell migration resistance across compartment boundaries due to initial ontogenetic tissue incompatibility – that is, cancer can only invade ontogenetically close tissues at first and acquires the trait to invade ontogenetically distant tissue later on – the reason for the migration resilience may become apparent. The bladder compartment is ontogenetically close, whereas the ureter and rectum compartment are ontogenetically far from the uterine cervix compartment. This translates then to a lower hurdle for cervical cancer to acquire the ability to invade the bladder compartment, but a profound step in tumour progression for the rectum and ureter compartment. The patterns of cervical cancer growth that we observed can be mapped to the tissue domains that are identified using the bifurcational developmental paradigm. Crucially, there is a clear correlation between the degree of ontogenetic proximity to the uterine cervix and the likelihood of infiltration by cervical cancer for any pelvic tissue. Indeed, when introducing ontogenetic distance on the tissue tree into our model of local tumour spread, the predicted step-wise tumour infiltration of the various pelvic tissue compartments is nearly identical to the ones observed in clinical practice, as seen in Fig. 4. In contrast, if the isotropic (microenvironmentally influenced) tumour growth model were true, the tumour infiltration probability of different pelvic structures should follow a (quasi-)stochastic process, i.e. the invasion probabilities of two compartments adjacent to the uterine cervix should be independent. Our findings do not support this notion, however. For example, the invasion probabilities of the rectal and ureteral compartments are practically zero if the bladder compartment is not infiltrated, indicating a statistic interaction (e.g. conditional probabilities) between these different infiltration likelihoods.

As a last step, we sought to corroborate our simulation findings concerning the *stepwise* tumour progression which we observed in the ontogenetic tumour progression model. To confirm that ontogenetic tissue compartment infiltration occurs in a predictable and step-wise fashion, tumour infiltration data was gathered by pathological examination of the surgical resected tissues and area-proportional Euler diagrams of the histopathological data of tumour infiltration were drawn (Fig. 5).

Our null-hypothesis of tumour invasion and transmigration based on microenvironmental cues would have predicted that there is a notable probability that the rectum or ureter compartment is infiltrated without infiltration of the bladder compartment. In fact, even by pure chance of probabilistic tumour growth, we would expect to observe a significant number of such tumour situations. Considering its proximity to the uterine cervix, the circumstance that the ureter is spared from tumour infiltration in most of the cases is by itself remarkable. This is not in support of the notion that microenvironmental factors alone determine tumour growth and invasion. Strikingly, except for one case, the rectum (mesorectum, muscular or mucosal layer) was never infiltrated without simultaneous infiltration of the bladder mesentery which strongly suggests a stepwise tumour progression based on tissue compartments with specifically different risks of tumour infiltration. Interestingly, in the one patient with rectum but without bladder infiltration, the bladder was spared during the initial surgery. After six months, this patient developed a tumour recurrence in the bladder compartment, indicating probable occult involvement of the bladder at the time of the initial operation. The nearly perfect sub-set characteristic of Fig. 5 can only occur if and only if the tumour progression happens in a stepwise fashion as our ontogenetic model would predict. Further information about the anatomical structures described here can be found in the Methods section.

One significant strength of the present work is that it is based on the histopathological data of numerous patients with advanced disease who underwent surgery without any preoperative treatment. Because patients with locally advanced disease are usually submitted to primary chemo-radiotherapy in most institutions, there is generally no comprehensive pathological data available. In a previous series of 88 cases with locally advanced and/or recurrent cervical cancer, the bladder with its support structures showed tumour infiltration in 84%, the rectum in 34% and the ureter in 24% of all cases^14^. In the current analysis examining 518 cases of primary cervical cancer, these patterns were slightly different with the ureter and mesureter exhibiting a greater likelihood of being infiltrated than the rectum and mesorectum. This demonstrates that tumour growth is different in cases of recurrent cancer as previous treatment (e.g. pelvic surgery or irradiation) probably affects ontogenetic compartment boundaries leading to different tumour progression patterns.

There are some weaknesses of our study which should be addressed. First, we used 2-dimensional anatomical maps instead of 3-dimensional anatomic templates. This was due to computational limitations and limited knowledge of the exact 3-dimensional positional information of all pelvic fascia. However, given the fact that the adjacent pelvic planes (sagittal and axial) are nearly identical to the ones we used, 3-dimensional simulation should lead to similar results.

Second, we disregarded other than continuous modes of tumour spread, i.e. lymphatic and haematogenous cancer dissemination. Although these routes of tumour propagation are clinically highly significant and have an important impact on a patient’s prognosis, we did not cover them, as our goal was to provide a roadmap for local tumour growth only. Furthermore, constructing precise anatomical templates of the lymphatic and circulatory system as a basis for modelling is currently not feasible. However, considering that lymph nodes receiving drainage from a specific anatomical region display antigens from that region^44^, they can be thought of as spatial extensions from that region providing a similarly fertile ground for tumour cell proliferation. We would speculate, that the knowledge about which tissue domain is connected to which lymph nodes could provide the basis for mapping the lymphatic landscape according to the display of antigens characteristic of specified ontogenetic compartments. Consequently, we would expect tumours to proliferate and spread preferentially to lymph nodes displaying antigens of close ontogenetic proximity to the peripheral tissue of tumour origin. Regarding hematogenic cancer spread causing metastasis in distant organs, we would again predict that organs which are ontogenetically closer related to the organ of tumour origin have a higher likelihood of being affected. However, the multitude of organs and anatomic structures sharing equal ontogenetic distance at this late stage of cancer progression makes it difficult to predict patterns of metastasis.

Third, this analysis only includes cervical cancer cases and its applicability to other tumour entities is therefore limited. However, the same principles regarding ontogenetically determined tumour growth seem to apply to vulvar cancer as was demonstrated in a recent clinical trial^45^. Furthermore, the correlation of carcinogenesis and ontogenesis has been shown for various tumor entities, such as rectum^46^, pancreas^47^, and mid-facial skin cancers^48^, wherein the local tumor spread can be well-described with embryological structures such as compartments and fusion planes.

Our findings provide a framework to conceptualize and predict local spread of cervical cancer. As ontogenetic compartment borders can clearly be identified intraoperatively, ontogenetic tissue mapping can probably serve as a roadmap to local cancer growth in other tumour entities. Knowing the embryological origin of any tissue in which cancer arises, one can derive a relative likelihood of different adjacent tissues to be infiltrated by tumour cells, depending on their ontogenetic proximity to the tissue of cancer origin, deduce an ontogenetic tumour stage, and tailor surgical resection exactly to the tissue at risk. In the future, this might help to decrease operative morbidity by sparing low-risk tissue while improving local tumour control by resecting all tissues of potential (occult) tumour infiltration.

What could be the underlying mechanisms on a cellular and molecular level? One explanation might be the existence of transcriptional programs which sequentially activate and silence specific genes. Such programs are executed by gene regulatory networks (GRNs), which are defined as units of interacting transcriptional elements which exhibit specific regulatory relationships and therefore cause a predictable pattern of gene expression with a defined structural and functional output^49^. GRN’s enable cells to concertedly change protein expression patterns in response to environmental changes and have a tendency to stabilize themselves including pathological states which might foster tumour progression^50^. This would lead to a predictable evolution which is reflected by the acquisition of all hallmarks of cancer on a cellular level and by specific growth patterns on the tissue level. Interestingly, GRNs seem to play an important role in epithelial-mesenchymal transition (EMT) which is a key mechanism of cell and tissue differentiation, both during ontogenesis and during cancer progression^51–53^ including hybrid epithelial/mesenchymal states^54–56^. We hypothesize that the stepwise re-activation of GRNs, which are normally executed during ontogenetic development, leads to the observed growth and infiltration patterns.

In conclusion, we have shown that the potential local tumour growth of cervical cancer is not isotropic. The data representation and our tumour growth simulation indicate that ontogenetic tissue mapping might profoundly help in predicting the probability of tumour invasion into a given tissue by a certain cancer. Future work needs to corroborate these findings, ideally in other cancer entities.

## Methods

### Patient selection and data acquisition

**Figure 6:**
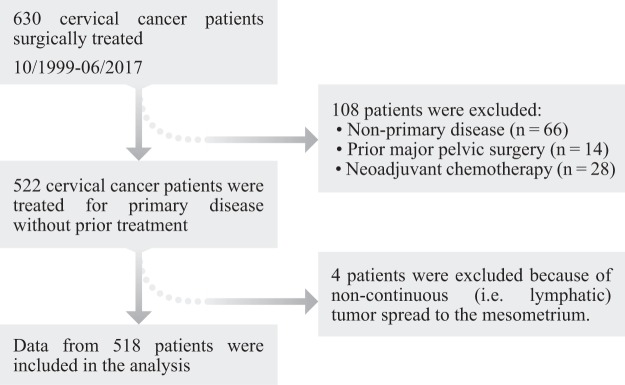
Patient selection process. Initially, 630 patients were enrolled and surgically treated between October 1999 and June 2017 for cervical cancer. Only patients with primary disease and without prior major pelvic surgery were included in the analysis. Four cases were excluded because of locally non-continuous tumour spread. Locally discontinuous tumour spread can occur to the mesometria via lymphatic vessels. Discontinuous local tumour spread to other tissues is anatomically not feasible. Additional information regarding the patient selection process and treatment is available in the Supplement S1.

In order to obtain histopathological information for further analysis, patient and tumour data was extracted from our institutional study databases. Figure 6 gives an overview over the patient selection process. More information regarding patient selection and treatment can be found in the Supplement S1. The TMMR/EMMR/LEER-Trial (cancer field resection trials) was approved by the ethical committee at the medical faculty of Leipzig University (review board numbers 012 – 13 – 28012013, 192/2001 and 151/366 2000). All aspects of the clinical trial as well as data handling and analysis was carried out in compliance with the relevant guidelines and directives issued by the European Union, the Federal Republic of Germany and the State of Saxony. The patients had given informed consent to participate in this study prior to the operation. This consent included usage of the data for further analysis. As the raw data contains sensitive patient information, it cannot be made accessible to the public. Researchers who meet the criteria to access confidential patient data will be supplied with the requested data on request. Further, non-sensitive data is available in the Supplement S1.

Numerical and qualitative data used for preparation of the Euler diagrams and in-silico models were extracted from the original detailed pathology reports archived in our database. These pathology reports contained all relevant information regarding tumour entity (i.e. squamous or non-squamous histology), tumour size measured directly within the surgical specimen, and extent of infiltration into neighbouring pelvic tissues.

### Ontogenetic anatomy

The terms we use to describe pelvic anatomy diverge from the terminology commonly used by gynaecological surgeons. Generally, structures and landmarks in surgical anatomy consist of functional units while “non-functional” connective tissue is frequently not classified any further. In contrast, ontogenetic anatomy provides a means to group all tissues into distinct groups regardless of form and function. Therefore, some of the terms which are less commonly used in conventional surgical anatomy or which have different meanings are described here. Figure 1 gives an anatomic overview.

The Müllerian compartment is the sum of all adult tissues which are derived from the embryonic paramesonephric ducts. Cranio-caudally, this includes the Fallopian tubes, the uterine corpus, the uterine cervix, the proximal two thirds of the vagina and the Müllerian adventitia (see below). Each of these structures constitutes a sub-compartment of the Müllerian compartment.

The para tissues constitute together the Müllerian adventitia. This is a coat of fibrovascular connective tissue enveloping the entire Müllerian compartment. In a cranio-caudal fashion the Müllerian adventitia can be divided into the following segments according to the adjacent organs:

- Fallopian tubes – parasalpinx
- Uterine corpus – paracorpus
- Uterine cervix – paracervix
- Vagina – paracolpos

Each para-tissue constitutes a sub-compartment of the Müllerian compartment.

Note that the term “parametrium” in surgical anatomy conventionally denotes a poorly defined composite structure including more tissues of different ontogenetic origin (paracervix, mesometrium, parts of the mesobladder and the mesureter). The para-tissues contain a tight network of anastomosing arteries and veins as well as lymph vessels and nerves.

The term mesentery is here applied to the connective tissues which connect the pelvic organs to the pelvic and abdominal wall laterally and caudally (and to a lesser extend ventrally in the case of the urinary bladder). The mesenteries are composed primarily of fibrofatty tissue containing lymph- and blood vessels and therefore serve an important nutritional role. In addition, they contain nerves and interspersed condensations of fibrous tissue which serves a suspensory function. Ontogenetically, they are closely related to the organs which they support and are therefore prone for early tumor infiltration. The mesenteries develop and elongate during the embryonic and fetal period as the associated organs move within the abdominal and pelvic cavity as a consequence of differential growth.

Within the pelvis, the urogenital tract is supported by the urogenital mesentery. This mesentery can be subdivided into

- The mesometrium supporting the uterus (corpus and cervix)
- The mesureter supporting the ureter
- The mesobladder supporting the urinary bladder.

In addition, the rectum is supported by the mesorectum.

By conventional nomenclature, only the mesorectum is a proper mesentery (defined as a peritoneal duplication enveloping an intraabdominal organ). From an ontogenetic perspective, the term “mesentery” is broader to include the above-mentioned structures.

### Anatomical and ontogenetic mapping

**Figure 7:**
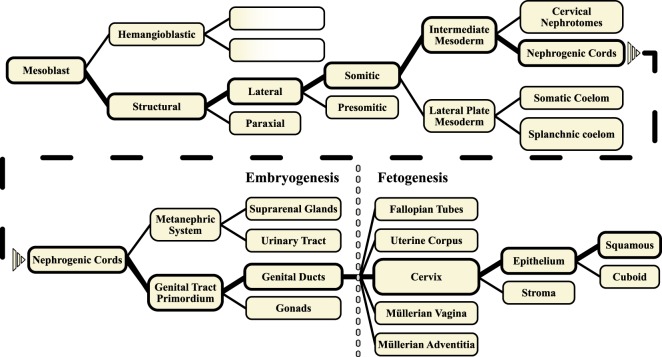
Ontogenetic tissue tree conceptualizing the development of the genital ducts sub-compartments (including the uterine cervix). For example, the nephrogenic cords give rise to the metanephric system and the primordial genital tracts. The primordial genital tracts then bifurcate to produce the gonads and the genital ducts (in the female, these are the Müllerian ducts). The lower Müllerian ducts fuse and develop into the mature reproductive structures including the fallopian tubes, the uterus, the uterine cervix and the proximal (Müllerian) vagina, which all constitute ontogenetic sub-compartments of the adult Müllerian compartment. The diagram depicted can serve as a genealogical tree to trace the ontogenetic development of a given tissue and to deduce the ontogenetic kinship of adjacent tissues. Note that in the post-embryonic (foetal) period indicated by the grey-dashed line, divisions into more than two tissue branches are possible. Contrary to the embryonic period, these developmental steps involve tissue differentiation along defined axes, e.g. craniocaudally without the establishment of new compartments.

We have previously described embryogenesis in terms of bifurcational tissue differentiation based on morphologic observations in human embryos^12,13^. During the first stage of tissue expansion in a given embryonic domain, cellular proliferation is homogenous and isotropic. At a certain point, presumably when a morphogen gradient is formed, and/or an unstable critical concentration is reached, cellular segregation and the emergence of two new cell populations with new phenotypes can be observed. Figure 7 shows the resulting bifurcational ontogenetic tissue tree in which the ancestry of different tissues can be traced. This tissue tree is the basis for the ontogenetic mapping shown in Fig. 1D.

### Modelling

As described in the beginning, singular tumours are usually not of spherical shape and do not necessarily grow isotropically. Nevertheless, permeation of cancer cells into surrounding tissues is assumed isotropic in order to define a safe surgical resection margin, hence they are assumed to have an isotropic growth potential. Here, we introduce a description of tumour growth dynamics for a *statistically averaged* tumour to show that the clinically assumed isotropic growth is not sufficient for explaining the clinically and pathologically observed tumour growth patterns. The descriptive model is based on the pathological and ontogenetic characterization presented Table 1 and Fig. 7 and is functional for the presented histological cancer types.

The growth dynamics for a statistically averaged tumour were described by the following set of reaction-diffusion equations

$${\partial }_{t}{\varPsi }_{ij,k}=\nabla \cdot ({D}_{ij,k}\nabla {\varPsi }_{ij,k})+{R}_{ij,k},$$

where ${\varPsi }_{ij,k}={\varPsi }_{ij,k}(\overrightarrow{x},t)$ denotes the probability density of cancer cells originating from tissue compartment *i* located in the tissue compartment *j* at a given time. The index *k* accounts for the cancer cell’s coarse-grained aggressiveness or staging, and ${D}_{ij,k}={D}_{ij,k}(\overrightarrow{x},t)$ denotes the diffusion coefficient of cancerous cells originating from compartment *i* at the stage *k* in the compartment *j*. The local cancer net proliferation rate ${R}_{ij,k}$ as a bulk may depend, for instance, on the complex cancer cell interactions with its environment. For example, increasing cancer cell density may lead to limited oxygen and nutrient supply per cell and therefore to a reduction in cancer cell division rate, or may lead to tumour hypoxia induced angiogenesis, thus stimulating tumour growth and invasion^57–62^. Some or many of inhibitory factors may lose effect in the progress of tumour progression^63–65^, thus altering the cancer proliferation and spreading dynamics. *In vivo*, the local cancer proliferation rate is generally thought to depend on supply of oxygen and nutrients per cancer cell, which is an inverse measure of the local cancer cell density, among potential other physical and chemical microenvironmental factors^42,66–70^. The reaction term can be expanded to include features of nutrient supply, microenvironmental effects or growth factors to create a model closer resembling *in vivo* tumour physiology and allowing for enforced anisotropic tumour growth. Here, the basic tumour growth rate was modelled by a generalized Fisher-Kolmogorov term^71–73^ agnostic of microscopic interactions of single cancer cells,

$${R}_{ijk}={r}_{ijk}{\varPsi }_{ik}(1-{\varPsi }_{ik}^{n}),$$

with the individual cancer cell proliferation rate *r*_*ijk*_ (as compared to *R*_*ijk*_) and the proliferation inhibition coefficient $(1-{\varPsi }_{ik}^{n})$. The cancer net proliferation rate *R*_*ijk*_ initially increases with an increasing number of cancer cells in a given volume and slows down when it approaches the maximum cancer cell density probability $\sum _{ik}{\varPsi }_{ik}=1$ in that volume. Tissue compartment boundaries were modelled as additional passive resistive barriers which limit cancer cell migration,

$$\hat{{\rm{n}}}\cdot (D\nabla {\varPsi }_{ij,k})=({\varPsi }_{j}-{\varPsi }_{i}\,)/{\rho }_{ij,k},$$

with $\hat{{\rm{n}}}$ denoting the normal vector of the interface of neighbouring compartments *i* and *j*, and *ρ*_*ij*_ the resistance for cancer cell migration across the interface. The resistive barrier simulates an initially low fraction of cancer cells with the acquired capacity of invasion into a neighbouring compartment. The finite-element model was simulated via COMSOL Multiphysics® software^74^. Further detailed descriptions and values of model parameters are given in the Supplement S2.

## Supplementary information

Supplement

## Data Availability

As the raw patient data of the TMMR/EMMR/LEER-Trial (cancer field resection trials) contains sensitive patient information, it cannot be made accessible to the public. Researchers who meet the criteria to access confidential patient data will be supplied with the requested data on request. Further, all non-sensitive data is available in the methods section and the supplemental information.
