# Supplementary material for: Roadmap to Local Tumour Growth: Insights from Cervical Cancer
Source: Sci Rep. 2019 Sep 4;9:12768. doi: 10.1038/s41598-019-49182-1 (PMC6726627; doi:10.1038/s41598-019-49182-1)
Supplement: Supplementary file 1 — Supplement [file 41598_2019_49182_MOESM1_ESM.pdf]

Supplement
Roadmap to Local Tumour Growth: Insights from Cervical Cancer

Hans Kubitschke[1+], Benjamin Wolf[2,3+], Erik Morawetz[1], Lars-Christian Horn[4], Bahriye Aktas[2], Ulrich Behn[5], Michael Höckel[2,3], Josef Käs[1*]

[1] Peter Debye Institute for Soft Matter Physics, Leipzig University, Germany

[2] Department of Gynecology, Women's and Children's Centre, University Hospital Leipzig, Germany

[3] Leipzig School of Radical Pelvic Surgery, Leipzig University, Germany

[4] Division of Gynecologic, Breast and Perinatal Pathology, University Hospital Leipzig, Germany

[5] Institute of Theoretical Physics, Leipzig University, Germany

S1 Patient selection

Since the beginning of the prospective monocentric studies evaluating the feasibility of total- and extended mesometrial resection (TMMR, EMMR) as well as laterally extended endopelvic resection (LEER) for the treatment of early (TMMR) and locally advanced (TMMR, EMMR and LEER) cancer of the uterine cervix, all clinical, surgical and pathological data was collected prospectively. The surgical procedures in TMMR [1], EMMR [2] and LEER [3] have been described before. As we sought to examine tumour growth unaffected by iatrogenic manipulations, we excluded all non-primary cases (i.e. tumour recurrences or secondary carcinomas). Furthermore, patients who had undergone extensive previous pelvic surgery (e.g. hysterectomy) or had received preoperative chemotherapy were excluded from the analysis as these treatments might affect tumour growth patterns. Because the objective of this study was to investigate continuous tumour spread, we further excluded cases of locally discontinuous (i.e. lymphatic) tumour spread. Locally discontinuous tumour spread can occur, to the mesometria via lymphatic vessels. Discontinuous local tumour spread to other tissues is anatomically not feasible. Pelvic lymph node status was assessed in all patients histopathologically. In 100 patients (19.3%) paraaortic lymph nodes were additionally examined histopathologically because of risk for metastasis to this region. However, we did not further analyse lymph node involvement as the present investigation focuses on local, continuous tumour spread. **Figure 6** of the manuscript gives an overview over the patient selection process. All patients had given written informed consent preoperatively which included permission to use data for further analysis. The TMMR/EMMR and LEER trials have been approved by the local ethics committee.

S2 Modelling

Simulations were carried out with COMSOL Multiphysics 5.3 (www.comsol.com). The set of partial differential equation are

$$\partial_t \Psi_{ij,k} = \nabla \cdot \left(D_{ij,k} \nabla \Psi_{ij,k} \right) + R_{ij,k} \,,$$

$$R_{ij,k} = r_{ij,k} \Psi_{ij,k} \left(1 - \Psi_{ij,k}^{n} \right) ,$$

$$\hat{\mathrm{n}} \cdot (D \nabla \Psi_{ij}) = \left(\Psi_j - \Psi_i \right) / \, \rho_{ij}$$

as described in the manuscript. The heat transfer module of COMSOL provided the simulation framework. Digitalized transversal planes were acquired from previous publications [4,1,3]. The simulations were done in two dimensions in the transversal plane. A summary of the simulation parameters for the physical-microenvironmental model are given in table 1 and for the ontogenetic model in table 2. For better comprehension, the parameter values here are given in *centimetres* and *days* instead of meters and seconds.

As boundary conditions, the tumour growth starting point is set to the centre of the cervix by imposing the tumour probability density to be $\Psi = 1$ at the external orifice of the cervix. The outer most boundaries of the transversal plane have a zero-probability flux normal to the boundary, meaning that no tumour can leave the body and the whole tumour entity is contained in the simulated transversal plane (the tumour cannot grow outside of the body).

The simulated transversal plane was rasterized into finite, trigonal elements and given differential equations were numerically solved. Initial parameters, e.g. dimensions of the pelvical area and tumour growth rates, were obtained from clinical findings given in the literature [5–10].

Table S1 Parameters for physical microenvironmental model

**Parameter**		**value**
general		
proliferation inhibition exponent		$n = 1$
diffusion coefficient		$c = 1.467 \cdot 10^{-4}\ \mathrm{cm}^2/\mathrm{d}$
finite element mesh properties		
maximum element size		1.00 cm
minimum element size		0.05 cm
maximum element growth rate		1.3
curvature factor		0.3
simulation time, time step		$0 - 1000\ \mathrm{d},\ \ 0.1\ \mathrm{d}$
Tissue characterisation	ontogenetic tissue type	Tumour growth rate
fibrous	cervix<br>paracervix	$r = 1.406 \cdot 10^{-2}\ 1/\mathrm{d}$
fatty	mesometrium<br>mesobladder<br>mesorectum<br>mesureter	$r = 1.875 \cdot 10^{-2}\ 1/\mathrm{d}$
muscular	bladder muscle<br>rectum muscle<br>ureter	$r = 0.469 \cdot 10^{-2}\ 1/\mathrm{d}$

The collagenous and ontogenetic boundaries are simulated as resistive barriers for tumour transgression with resistances given in table 2. The resistivity is perpendicular to the boundary. The ontogenetic compartments are illustrated in **Figure 1D** as grouped colours of same type. The collagenous lamellae are located between neighbouring compartments as described in Steineke et al [11]. The transmigration resistivity between two facing compartments in the ontogenetic model is based on the *distance* in the ontogenetic tree of the invaded compartment and the originating compartment of the tumour cells, given in **Figure 3**. The larger the distance the higher the resistivity. The resistivity is assumed to double for every major bifurcation. The finite-element model was simulatied via COMSOL Multiphysics® software [12]

Table S2 Parameters for onotegenetic model

**Parameter**		**value**
general		
proliferation inhibition exponent		$n = 1$
diffusion coefficient		$c = 1.467 \cdot 10^{-4}\ \mathrm{cm}^2/\mathrm{d}$
Tissue characterisation	ontogenetic tissue type	Tumour growth rate

fibrous	cervix<br>paracervix	$r = 1.406 \cdot 10^{-2}\ 1/\mathrm{d}$
fatty	mesometrium<br>mesobladder<br>mesorectum<br>mesureter	$r = 1.875 \cdot 10^{-2}\ 1/\mathrm{d}$
muscular	bladder muscle<br>rectum muscle<br>ureter	$r = 0.469 \cdot 10^{-2}\ 1/\mathrm{d}$
Migration from tissue i…	…into tissue j	Transmigration resistivity ρ_{ij}
Paracervix	Mesometrium	$0.217 \cdot 10^{3}\ \mathrm{d/cm^2}$
Mesometrium	Mesobladder	$0.434 \cdot 10^{3}\ \mathrm{d/cm^2}$
Mesometrium<br>Mesobladder	Mesureter<br>Ureter	$0.868 \cdot 10^{3}\ \mathrm{d/cm^2}$
Mesometrium	Mesorectum	$1.736 \cdot 10^{3}\ \mathrm{d/cm^2}$

S3 Step-wise Tumour Infiltration

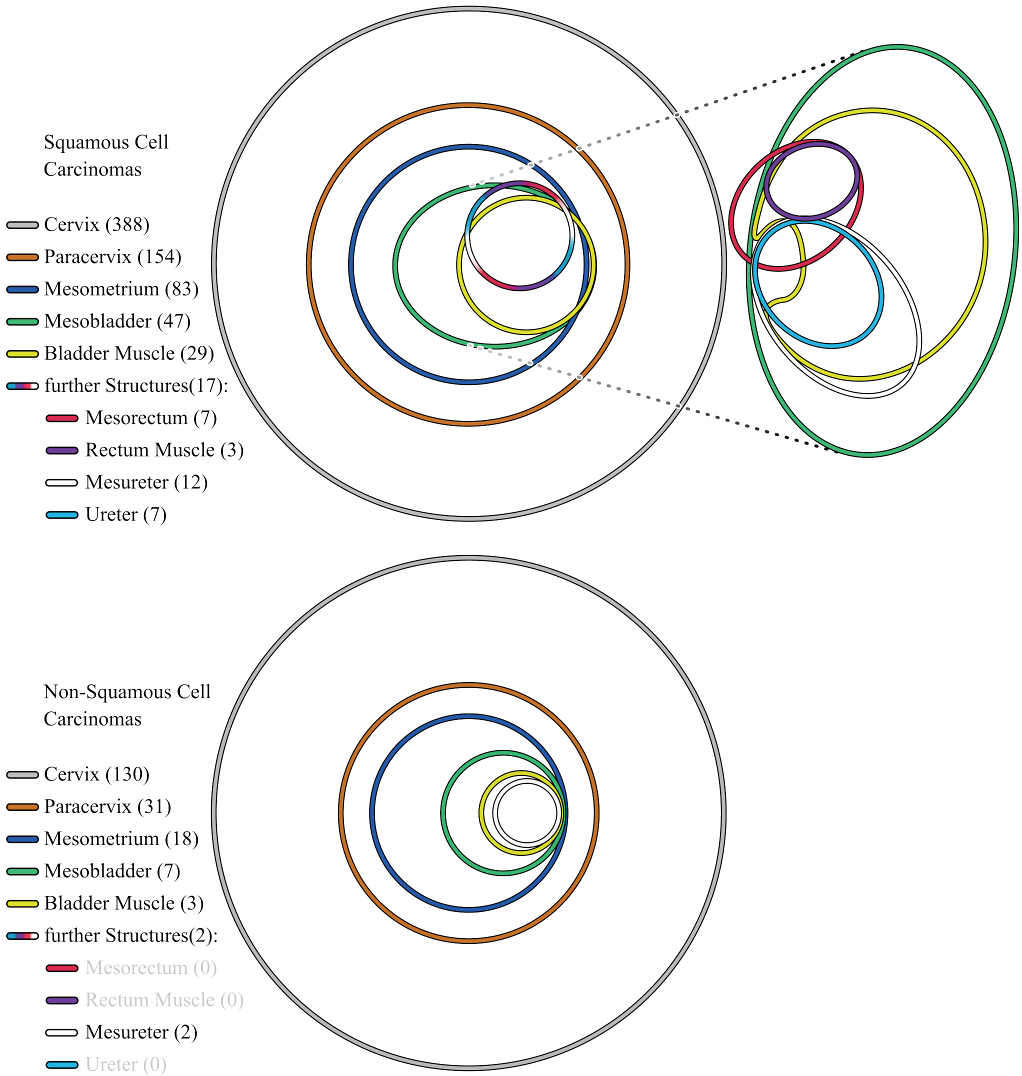

Figure S1: Separated Area-proportional Euler diagram of infiltration of endopelvic (sub-) compartments of 388 cases of squamous cell carcinomas and 130 cases of non-squamous cell carcinomas of the uterine cervix. Both sub-sets of the larger pooled set of 518 cases show a step-wise tumour infiltration with only a few exceptions.

S4 References

1. Höckel M, Hentschel B, Horn L-C (2014) Association between developmental steps in the organogenesis of the uterine cervix and locoregional progression of cervical cancer. A prospective clinicopathological analysis. The Lancet Oncology 15 (4): 445–456.
2. Wolf B, Ganzer R, Stolzenburg J-U, Hentschel B, Horn L-C et al. (2017) Extended mesometrial resection (EMMR): Surgical approach to the treatment of locally advanced cervical cancer based on the theory of ontogenetic cancer fields. Gynecologic oncology 146 (2): 292–298.
3. Höckel M, Wolf B, Hentschel B, Horn L-C (2017) Surgical treatment and histopathological assessment of advanced cervicovaginal carcinoma. A prospective study and retrospective analysis. European journal of cancer (Oxford, England : 1990) 70: 99–110.
4. Höckel M (2015) Morphogenetic fields of embryonic development in locoregional cancer spread. The Lancet Oncology 16 (3): e148-e151.
5. Zharinov GM, Gushchin VA (1989) Skorost' rosta opukholi i kletochnaia poteria pri rake sheĭki matki. Voprosy onkologii 35 (1): 21–25.
6. Combes PF, Douchez J, Carton M, Naja A. (1968) Etude de la croissance des métastases pulmonaires humaines comme argument objectif d'evaluation du pronostic et des effets thérapiques. Journal de Radiologie et d'Electrologie 49: 893–902.
7. Cosper P, Olsen JR, Siegel B, Dehdashti F, Schwarz JK et al. (2015) Cervical Tumor Volume Doubling Time. A Pilot Study. International Journal of Radiation Oncology*Biology*Physics 93 (3): E258-E259.
8. Harper LM, Odibo AO, Stamilio DM, Macones GA (2013) Radiographic measures of the mid pelvis to predict cesarean delivery. American journal of obstetrics and gynecology 208 (6): 460.e1-6.
9. Trott KR, Kummermehr J (1985) What is known about tumour proliferation rates to choose between accelerated fractionation or hyperfractionation? Radiotherapy and oncology : journal of the European Society for Therapeutic Radiology and Oncology 3 (1): 1–9.
10. Bolger BS, Symonds RP, Stanton PD, MacLean AB, Burnett R et al. (1996) Prediction of radiotherapy response of cervical carcinoma through measurement of proliferation rate. British journal of cancer 74 (8): 1223–1226.
11. Steinke H, Wiersbicki D, Speckert M-L, Merkwitz C, Wolfskämpf T et al. (2017) Periodic acid-Schiff (PAS) reaction and plastination in whole body slices. A novel technique to identify fascial tissue structures. Annals of anatomy = Anatomischer Anzeiger : official organ of the Anatomische Gesellschaft 216: 29–35.
12. COMSOL Multiphysics® v. 5.3. www.comsol.com. Stockholm, Sweden.
